# Retraction: TRB3 Is Involved in Free Fatty Acid-Induced INS-1-Derived Cell Apoptosis via the Protein Kinase C δ Pathway

**DOI:** 10.1371/journal.pone.0301945

**Published:** 2024-04-03

**Authors:** The PLOS ONE Editors

Following the publication of this article [1], concerns were raised regarding the results in multiple figures. Specifically,

- The Palmitate+ SiNC panel in Figure 3B appears to partially overlap with the Palmitate- U-73122+ panel in Figure 4B when rotated clockwise.
- The following panels appear to fully or partially overlap:
  - Figure 5G, Dox+ Palmitate+ panel, and Figure 5H, Dox+/Palmitate+ rotterlin- panel when rotated 180 degrees.
  - Figure 5H, Dox+/Palmitate- rotterlin- panel, and Figure 5G, Dox- Palmitate+ and Dox+ Palmitate- panels.
  - Figure 1B, 0mM panel and Figure 2C, Dox- Palmitate- panel.
  - TRB3 and β-actin panels in Figure 1G and Figure S3.

The corresponding authors stated that primary data underlying all the results reported in the article are available, except for underlying data for Figure 1G (TRB3 panel), Figure 3A, Figure S1, and Figure S3, which are no longer available.

In response to queries about these concerns, the corresponding author stated that the following panels are incorrect: Figure 1B, 0mM, 0.4 mM and 0.8 mM panels; Figure 3B, Palmitate+ SiNC panel; and Figure 5G, Dox- Palmitate+ panel. They provided replacement images for these panels and the Dox+ panels in Figure 5G, as well as image files for all other panels in these figures, images described as replicate samples for these experiments, and data tables underlying all graphs in these figures. Editorial assessment of the original data raised additional image concerns which undermine the reliability of these data, and the *PLOS ONE* Editors therefore consider the data insufficient to address the issues in these figures.

The corresponding author stated that the TRB3 and β-actin panels in Figure 1G and Figure S3 are intentionally identical. The Editors consider the concern about the reuse of these images resolved, but note that this should have been reported in the figure legends.

In light of the concerns affecting multiple figure panels and the underlying data that question the reliability of the published results, the *PLOS ONE* Editors retract this article.

JQ, LP, and LC did not agree with the retraction and stand by the article’s findings. NF, JLou, WZ, SX, HL, QF, ZW, JLiu, and XM either did not respond directly or could not be reached.
